# Self-sampling coupled to the detection of HPV 16 and 18 E6 protein: A promising option for detection of cervical malignancies in remote areas

**DOI:** 10.1371/journal.pone.0201262

**Published:** 2018-07-23

**Authors:** Kátia Luz Torres, Josiane Montanho Mariño, Danielle Albuquerque Pires Rocha, Mônica Bandeira de Mello, Heydy Halanna de Melo Farah, Renato dos Santos Reis, Valquíria do Carmo Rodrigues Alves, Edson Gomes, Toni Ricardo Martins, Ana Carolina Soares, Cristina Mendes de Oliveira, José Eduardo Levi

**Affiliations:** 1 Programa de Pós-Graduação em Ciências da Saúde da Universidade Federal do Amazonas (UFAM), Manaus, Amazonas/Brazil; 2 Programa de Pós-Graduação em Imunologia Básica e Aplicada da Universidade Federal do Amazonas (UFAM), Manaus, Amazonas/Brazil; 3 Fundação Centro de Oncologia do Estado do Amazonas (FCECON), Manaus, Amazonas/Brazil; 4 Universidade Federal do Amazonas—UFAM, Coari, Amazonas/Brazil; 5 Laboratório Sebastião Marinho, Manaus, Amazonas/Brazil; 6 Instituto de Medicina Tropical da Universidade de São Paulo, São Paulo/Brazil; Istituto Nazionale Tumori IRCCS Fondazione Pascale, ITALY

## Abstract

**Objective:**

To evaluate both the performance and acceptability of a method coupling self-sampling with detection of cervical malignancy via elevated HPV 16 and 18 E6 oncoproteins (Onco***E6***™ Cervical Test) in remote areas in Brazil.

**Methods:**

Women living in rural villages in proximity to Coari city, Amazonas, Brazil were invited to participate in a cervical cancer screening study. 412 subjects were enrolled; there were no refusals. In addition to E6 protein detection, DNA was extracted from the brushes and evaluated for HPV genotypes by PCR (PGMY09/11), followed by typing by the Papillocheck™ if positive. Subjects who were found to be positive for OncoE6 or HPV-DNA were referred for colposcopy.

**Results:**

For 110 subjects (27%) this was the first cervical cancer exam. Overall the HPV-DNA prevalence was 19.1% (n = 79); 1.4% (n = 6) were positive by the OncoE6 Test. Fifty-six women attended the invitation for colposcopy where nine had an abnormal cervix and were subsequently biopsied. Histopathological analysis revealed 2 CIN3, 2 carcinomas and 5 CIN1. OncoE6 called two out of the three HPV 16 or 18 associated CIN3+ lesions.

**Conclusions:**

The findings suggest that self-administered sample collection in combination with OncoE6 Test is feasible in this population. This could enable expanded screening coverage while ensuring a high specificity which is imperative given the remote geographic location, since women bearing abnormal test results would necessitate travel and logistical burden to access colposcopy and treatment.

## Introduction

In the last decade, large randomized controlled trials conducted in developed nations have demonstrated the superiority of HPV nucleic acids detection over cytology for primary screening for cervical cancer (CC) [1]. In contrast, Pap cytology has a high rate of false negative results, particularly for high-grade cervical malignancies; furthermore, the infrastructure requirements of prevention using Pap cytology frequently exceed capacity of low resource settings [2]. Several countries [3] (e.g. Australia, Italy, UK, Sweden, Norway, Canada and the Netherlands) are actively transitioning toward HPV tests in lieu of cytology as a first line screening strategy, given high sensitivity and cost-effectiveness. Consequently, rates of CC–at least in high income countries- are projected to decline.

Unfortunately, this may not be the case in low-income countries where access to cervical screening is not widely available. These neglected and unscreened/underscreened populations are at highest risk for CC [4], Independent of the screening approach, the coverage rate (i.e. access), remains a major public health challenge.

There are several reasons as to why women may not adhere to a CC screening program. Low level of education, with relative lack of awareness of the disease risk and prevention, coupled with limited access to any existing screening offers are two major contributing factors.

An innovative, active approach has been proposed: rather than adopting a passive approach whereby women come for screening, rather one should deliver screening to the women. One such approach is self-administered sampling (SS). SS has been shown to be as effective as collections by health-care workers for downstream HPV testing [5]. SS can also complement existing approaches e.g. targeting women who fail to attend their CC screening visits [6,7,8].

Several devices have been validated for the collection of cervicovaginal material for HPV nucleic acid testing (NAT). These devices are sent by mail to patients who -in general- respond favorably to the SS approach [7,8]. Self-administered sampling increases coverage by making testing available to women with limited or no access to the health system. In these instances, a health provider delivers the self-sampling device to women at–or proximate- to their homes, along with written or oral instructions for use, as well as submission to the lab for processing [9,10].

Brazil has an estimated incidence of 17 CC cases/100,000 women per year with important regional differences [11]. In the North region, comprising the Brazilian Amazon, CC is the leading cause of cancer in women (by incidence); it is subsumed by breast and colorectal cancers in the other regions of the country [11]. Geographic isolation, of those living in the North region, is thought to be a major contributing factor given obstacles to health care access.

The estimative for CC incidence for the Amazonas state and Manaus are 47 and 61 /100,000 women respectively [11], which is extraordinarily high (as a measure of comparison the incidence in the United States [12] is 8/100,000). Consequently, hundreds of cases of CC are detected each year in the state of Amazonas and referred to Manaus for oncological management. Fundação CECON is the only public oncology hospital in the state, and is burdened both by the high number of regional cases as well as those referred from neighboring states.

Prevention is both at a lower cost and more effective than treatment. Such is the case for CC whereby the current 5-year mortality rate from diagnosis is 50% in Amazonas state [13]. In recent years, screening coverage has been less than 60% of the target population. We sought to evaluate preventive strategies, so as to interdict early stage cancers, when cure is still achievable.

Over the last decade, economic initiatives by the Brazilian government have been implemented to assist impoverished families; this has contributed to the improvement of social conditions. A stipend is paid preferentially to eligible women. Eligibility contingent upon their children attending school and adherence to a vaccine schedule. In addition, women must attend the CC screening program. Despite this initiative, the regional incidence of CC has not decreased, in part due to limitations of the screening approach i.e. cytology [14]. Access to colposcopes and trained colposcopists, is an obstacle, whereby women with abnormal smears (e.g. atypical squamous cells of undetermined significance or higher (ASCUS+) require transportation to Manaus, a journey that can take from 8 hours to 20 days by boat, for colposcopic examination. This becomes logistically prohibitive. Furthermore, given low specificity of cytology in the context of ASCUS+, the majority of the colposcopies are negative, engendering resistance among women against repeat visits, and influencing healthcare decision-making among other women in their communities.

We aimed to evaluate an approach that would: increase populational coverage, detect CC and precursor lesions at a high sensitivity and, be as specific as possible, only referring women at high risk for CC. Providing a rapid feedback to the women was also seen as an extra but extremely valued asset.

## Materials and methods

We sought to investigate alternatives to the current, regional CC screening program, which uses cytological screening as recommended by the Brazilian Ministry of Health. Our focus is the interior of the Amazon State, which is the largest state in Brazil, covering a geographical area of 1,559,159,148 km^2^, equivalent to that of Germany, France, UK and Spain altogether. The population of Amazonas is 4 million people, half of whom reside in Manaus, the state capital.

Most inhabitants are subsistence farmers, living off from what they harvest and/or extract from the river. They are referred to as “ribeirinhas”. The local economy is based on fishing but in recent years there has been investment in Coari through natural gas exploitation. This has led to mass migration (e.g. workers) of predominantly men, to the area, increasing the incidence of sexually transmitted diseases.

Our strategy consisted of self-sampling followed by an immunochromatographic test that is simple to perform (the Onco***E6***™ Cervical Test, Arbor Vita Corporation, Fremont, CA, USA); Onco***E6*** detects the cancer causing virally encoded E6 oncoprotein of HPV types 16 and 18, which correlates to the presence of high-grade cervical lesions, as previously demonstrated in a triage study in Barretos [15], São Paulo state, Brazil.

The pilot study was conducted in the municipality of Coari, about 360 km from Manaus, navigable by the Solimões river. Coari is a 60,000-inhabitant’s city, half of which reside in the rural area in many communities of *ribeirinhos*. A local health agent visited the villages in advance, informing and explaining to women the purpose and procedures of the study, including self-sampling.

A team from FCECON coordinated the fieldwork, receiving logistical support from Coari municipality, i.e. provision of a boat, crew and food for research team use. The research team was composed of public health agents, undergraduate and graduate students from the Federal University of Amazonas (UFAM), researchers from FCECON and Instituto de Medicina Tropical da Universidade de São Paulo. This study was approved by the Ethical Committee from FCECON prior to initiation; subject enrollment was conducted under full informed consent.

From August 2014 to March 2015, 30 villages were visited by the research team: Itapéua, Isidório, Vila Lira, Saubinha, Esperança I, Ananidé, Nossa Senhora de Fátima, Nossa Senhora de Aparecida, Menino Jesus, Santa Maria, Nossa Senhora do Livramento, Laranjal, São José da Boa Vista, São Tomé do Patoá, Andirá, Lauro Sodré, São José do Estirão Sto Antonio, Nossa Sra Perpétuo Socorro Boa Fé, Santa Maria do Poção, Vila Fernandes, Santa Terezinha, Santo Expedito, Vila Trocaris, Amazonino Mendes, São Rdo da Costa do Trocaris, Nova República, São Pedro, Santa Rosa and Canaã. The participating villages are situated in rural locations and reachable by boat; the most remote village is located 60 km from Coari.

Enrollment was spontaneous. All women that presented to the research team at the day of the visit to a locality, and manifested their will to participate in the study were enrolled, upon signature of the informed consent form.

### Self-sampling

Women were offered self-sampling with Evalyn™ brush (Rovers, Netherlands). Detailed, illustrated instructions, previously explained by a health agent, was provided to the candidates with the device. Samples were collected at home or in the local schools and health centers. Brushes were transported to Laboratório de Genética do Instituto de Saúde e Biotecnologia de Coari da Universidade Federal do Amazonas in Coari, where the OncoE6 test (Arbor Vita Corporation, Fremont, CA, USA), DNA extraction and HPV generic PCR were performed. Maximum time in between sample collection and processing was 96 hours, during which samples were stored at 4°C.

### OncoE6

Caps were removed from brushes and bristles were cut with a disposable scalpel and placed in a microtube. Samples were lysed with a reagent contained in the kit and centrifuged at 10,000xg for 10 minutes. An aliquot of 200μL from the supernatant was incubated again with the lysis and conditioning solutions, containing high-affinity monoclonal antibodies against the protein E6 from HPV 16 and 18 conjugated with alkaline phosphatase(AP). Following a 10-minute incubation the solution was pipetted into individual cartridges containing the same monoclonal antibodies anti-E6 from HPV 16 and 18 spotted into a nitrocellulose membrane in separate bands. The lysate was allowed to migrate over the strip for 55 minutes and further processed for color development employing AP substrate for another 10 minutes.

### DNA extraction

All excess supernatant and bristles were removed and the cellular pellet was re-suspended in 200 μL of DNAase free ultrapure water (Thermo Fisher, São Paulo, Brazil). The suspension was submitted for DNA extraction using the Qiagen DNA Mini kit (Qiagen, São Paulo, Brazil) in accordance with the manufacturer instructions.

### Generic L1 HPV DNA testing

50–250 ηg of extracted DNA was added to a PCR mixture containing PGMY09/11 primers [16] spanning a 450 bp fragment from L1 gene of mucosal HPVs in addition to primers targeting human β-globin gene (268 bp) at 200ηM each, 4mM MgCl_2_, dNTPs 200ηM, glycerol 5.7% and Cresol Red 250 ηg/μL. PCR products were run on a 2% agarose gel, stained with ethidium bromide and visualized under UV light irradiation.

### HPV Genotyping (Papillocheck™, Greiner Bio-One, Frickenhausen, Germany)

An aliquot of the DNA obtained from all samples demonstrating a band of 450bp in the generic PCR, as above, was subjected to microarray HPV genotyping at the Virology lab from the Instituto de Medicina Tropical da Universidade de São Paulo (São Paulo, Brazil); the latter is based on PCR amplification of a fragment of approximately 350bp from the E1 region of HPV genomes using broad-spectrum consensus primers. Amplicons are hybridized to type-specific probes immobilized on plastic slides, allowing simultaneous detection and genotyping of 24 different HPV types (HPVs 16,18,31,33, 35, 39,45,51,52,53,56,58,59,66,68,70,73,82), including 6 low-risk HPVs (6,11,40,42,43,44).

### Patient follow-up

Women who were found to be reactive either by OncoE6 or generic L1 HPV PCR were offered colposcopy. A trained colpolscopist from Manaus (MBM) performed the colposcopy in Coari.

## Results

A total of 412 women agreed to participate; the age distribution and socio-demographic characteristics are presented in Table 1. Parity index is high (Table 2), One-third of the women had ≥five children; attributable to lack of access to information and contraceptive measures, including refusal of condom use by male partners.

**Table 1:** Socio-demographic characteristics, study participants (N = 412), ribeirinhas women from the municipality of Coari–Amazonas state, 2014–2015.

Characteristic	N (%)
**Ethnicity (self-assessment)**	
*“Parda”*^*&*^	368 (89,3)
White	34 (8,3)
Black	9 (2,2)
Indian	1 (0,2)
**Age yo**	
18–25	79 (19,1)
26–36	150 (36,4)
36–46	99 (24)
46–54	39 (9,4)
≥ 54	45(10,9)
**Civil state**	
Single	41 (10)
Married	132 (32)
Stable union^**@**^	216 (52,4)
Divorcied	5 (1,2)
Separated	5 (1,2)
Widow	13 (3,2)
**Education**	
Illiterate	35 (8,5)
1–5 years	220 (53,4)
6–10 years	72 (17,4)
>10 years	85 (20,6)
**Ocupation**	
Farmer	321 (78)
Housewife	17 (4,1)
Student	13 (3,2)
Governmental	43 (10,4)
Unemployed	3 (0,7)
Retired	13 (3,2)
Other	2 (0,4)
**Income (monthly)**	
1 salary*	128 (31)
2–3 salaries	29 (7)
*“Bolsa-família”* ^#^	255 (61,8)
**Total**	**412 (100)**

* In Brazil is common to use the value of the minimal salary, established by the federal government, as a reference. In the time of the study 1 salary ≈ 250,00 USD.

# “*Bolsa-família”* is a salary paid by the government to families living below the poverty line. Values vary according to the number of children at school and other situations, on average ≈ 150,00 USD/month.

& Parda is the word used in official forms for the assessment of ethnicity in Brazil. A synonym would be the word “mullato”.

@ Stable Union is the legal term for all couples that are not married but have lived together time enough to confer to their relationship the same legal status as an official marriage.

**Table 2:** Behavioral characteristics of the study population, *ribeirinhas* women from Coari/AM, 2014–2015 (N = 412).

Variable	N (%)
**Sexual debut**	
≤ 15 yo	162 (39,3)
˃ 15 yo	250 (60,7)
**Parity (children)**	
0	23 (5,6)
1	55 (13,4)
2–5	169 (41)
5–10	132 (32)
>10	33 (8)
**Condom use**	
Never	179 (43,4)
Sometimes	161 (39)
Always	72 (17,4)
**Sexual partners last year**	
0	33 (8)
1	363 (88,1)
2–5	15 (3,7)
>5	1 (0,2)
**Sexual partners lifetime**	
1	164 (39,8)
2–4	180 (43,7)
5–10	52 (12,6)
> 10	16 (3,9)
**Abortions**	
< 1	283 (68,7)
≥ 1	129 (31,3)
**Use of contraceptives**	
Yes	164 (39,8)
No	248 (60,1)
**Smoking history**	
Yes (current smoker)	63 (15,2)
Yes (quit)	78 (18,9)
No	271 (65,7)
**Alcohol comsumption**	
Yes	5 (1,2)
No	407 (98,8)
**Pap smear history**	
Never	110 (26,7)
Every year	241 (58,5)
Every 2 years	37 (9)
Sporadically	17 (4,1)
Unknown	7 (1,7)
**History of Sexually Transmitted Diseases**	
Yes	14 (3,4)
No	398 (96,6)

In general, self-administered sampling had high level of acceptance, with only 2% (N = 9) disapproving of the procedure; 95% considered it simple to perform and 80% preferred this mode of collection than by a health professional.

Over the course of the study, seven visits to the villages were conducted, each taking from 1–2 days; samples were maintained dry, and refrigerated (2–8°C) in the boat for a maximum of 4 days, prior to delivery at the laboratory in Coari. Upon arrival, samples were always processed on the same day.

### L1 PCR

All samples showed proper amplification of the human β-globin fragment, attesting the adequacy of the collection procedure. DNA from seventy-nine samples (18.6%) amplified the 450 bp band and were considered HPV-DNA positive, including the six OncoE6+ (see below).

### HPV genotyping

The 79 HPV+ DNAs were subjected to the Papillocheck™ assay; 46 were positive of which HPV 51 was the most prevalent (n = 8), followed by HPV 16 (n = 7), HPV 53 (n = 5), HPV 18, 31 and 70 (n = 4, each), HPV 52, 56, 66 and 82 (n = 3, each), HPV 43, 58 and 68 (n = 2, each) and HPV 11, 40, 45 and 59 (n = 1, each). The sum surpasses 46 due to co-infections which were observed in 14 patients out of the 46 with detectable HPV DNA. Fourteen samples were negative, despite being HPV L1 positive, and 19 were found invalid following failure to amplify the endogenous gene (ADA) included in the Papillocheck method.

### Colposcopy

Seventy-nine HPV-DNA positive patients were invited to undergo colposcopy, when a liquid-based cytology specimen was collected (SurePath, BD, Brazil).

Fifty-six completed the procedure while one patient didn`t collect a liquid-based cytology sample. Macroscopic cervical abnormalities were detected in 9 who were biopsied (Table 3).

**Table 3:** Colposcopy and histology results.

	N =	(%)
NOT REFERRED TO COLPOSCOPY (HPV L1 PCR -)	333	81
REFERRED TO COLPOSCOPY (HPV L1 PCR/ONCO E6 +)	79	19
DIDN`T ATTEND/REFUSED COLPOSCOPY	23	28
COLPOSCOPY RESULTS	56	72
Normal	47	84
Abnormal (Biopsied)	9	16
CIN 1	5	56
CIN 3	2	22
CARCINOMA	2	22
TOTAL	412	100

### Liquid-based cytology and histopathology

Forty-one smears were classified as Negative for Intraepithelial Lesion or Malignancy (NILM), 7 as Atypical Squamous Cells of Undetermined Significance (ASCUS), 5 were low-grade squamous intraepithelial lesions (LSILs) and 2 high-grade squamous intraepithelial lesions (HSILs). Of the nine biopsies, five had evidence of cervical intraepithelial neoplasia grade 1 (CIN1), two CIN 3 and two had invasive carcinomas (one squamous and the other adenosquamous) (Tables 3, 4 and 5).

**Table 4:** Colposcopy and laboratory results from the 6 OncoE6+ patients.

Onco E6^TM^ Positives	CYTOLOGY	COLPOSCOPY	HISTOLOGY	PCR PGMY09/11	GENOTYPES PAPILLOCHECK
HPV 16 N = 3	HSIL	ALTERED	CARCINOMA	POSITIVE	HPV 16
	LSIL	ALTERED	CIN 3	POSITIVE	HPV 16
	NOT DONE	NOT DONE	NOT DONE	POSITIVE	HPV 16
HPV 18 N = 3	HSIL	ALTERED	CIN 1	POSITIVE	HPV 18, HPV 68
	NILM	Normal	NOT DONE	POSITIVE	HPV 18, HPV 51
	LSIL	Normal	NOT DONE	POSITIVE	HPV 18

**Table 5:** Profile of the CIN1+ cases.

Onco E6™	PCR(PGMY09/11)	Cytology	Colposcopy	Histology	Genotyping
HPV 18	POSITIVE	HSIL	ALTERED	CIN 1	HPV 18
NEGATIVE	POSITIVE	LSIL	ALTERED	CIN 1	HPV 51
NEGATIVE	POSITIVE	NILM	ALTERED	CIN 1	HPV 31
NEGATIVE	POSITIVE	NILM	ALTERED	CIN 1	HPV 44, HPV 56
NEGATIVE	POSITIVE	NILM	ALTERED	CIN 1	UNTYPED
NEGATIVE	POSITIVE	NILM	ALTERED	CIN 3	HPV 45, HPV 73
HPV 16	POSITIVE	LSIL	ALTERED	CIN 3	HPV 16
HPV 16	POSITIVE	HSIL	ALTERED	SQUAMOUS-CELL CARCINOMA	HPV 16
NEGATIVE	POSITIVE	NOT DONE	ALTERED	ADENOSQUAMOUS CARCINOMA	HPV 18#, HPV 39 HPV 56, HPV 81

# Tumor tissue was positive exclusively for HPV 18 by the Papillocheck and DNA sequencing

### OncoE6

Six samples (1.4%) were found positive for this assay, 3 for HPV 16 E6 and 3 for HPV 18 E6 (Table 4). The overall clinical sensitivity for CIN 3+ was 2/4 = 50% if the analysis is not restricted to HPV 16 and 18, the unique genotypes targeted by this method, since there was one CC case harboring HPV 45 (Table 5). The clinical specificity was 99% (408/412). All six OcoE6 reactive samples were also positive for the L1 PCR and the genotyping assay confirmed the presence of HPV 16 and HPV 18 respectively on all, being totally concordant. Four women harbored HPV 16 DNA and one HPV 18 DNA without concomitant detectable E6 protein.

### Patient follow-up

CIN 1 cases were followed-up at the health unit in Coari, where they were counselled to repeat the examination in one year. The two CIN 3 and two ICCs were referred to FCECON in Manaus for definitive management. DNA was extracted from the the adenosquamous carcinoma after conization surgery and submitted for real-time PCR assay specific for HPV 18; this confirmed positivity and also revealed a single HPV 18 infection when genotyped by the Papillocheck.

## Discussion

Over the course of the last two decades, Brazil has transitioned from a low to an upper middle-income country [17]. This is evidenced by an improvement in socio-economic and key health indicators such as life expectancy and childhood mortality rate. Advances in health have not extended to cervical cancer, a preventable neoplastic disease. Consequently, incidence of cervical cancer remains high, reflecting deficiencies in extant prevention/screening programs.

There are several reasons for the deficiencies of the current screening program. Foremost, low screening coverage of the target population is a major factor that underlies the failures of the CC screening program. In contrast, some women are over-screened, undergoing annual Papanicolaou testing, contrary to the Brazilian guidelines that recommends a three-years interval. This serves to deplete, limited resources that could otherwise be directed toward expanding access to those in greater need [18]. Second, is the cytology diagnosis itself, given frequent under-representation of the squamo-columnar junction and false-negative results [14]. Third, delays prior to colposcopy, and reporting of histologic results, impact treatment and increase probability of invasion even if they may have been contained at time of diagnosis.

Our results showed the incidence of CC to be even higher than the estimates; 2 cases were observed in 412 women, making a raw, non age-adjusted incidence of 485/100,000! Two high-grade precursor lesions (CIN3) were detected and treated, potentially avoiding another two ICC cases. Since laboratory processing is quite simple for the OncoE6 assay, samples were able to be processed and tested at a low-technology local laboratory with reporting of results within a few days of collection. By contrast, these women reported Pap smear waits of up to a year prior to resulting.

HPV NAT have been transformative given their high sensitivity to detect high-grade cervical abnormalities, yet low clinical specificity given that the majority of HPV+ women have normal cervices. In the remote geographic setting such as in our study, specificity is a high priority, given the logistic challenges of follow-up. Low specificity in CC screening can also lead to overtreatment, which may impair reproductive capacity of those submitted to surgical interventions [19]. To this end, a specificity of 99% is a key strength of our approach.

Sensitivity is central to any cancer screening test. OncoE6 missed 2 out of the 4 CIN3+ cases. One case had a NILM cytology and harbored HPV 45, a genotype not included in the version of the OncoE6 assay employed. Consequently, if we restrict the analysis to CIN3+ cases driven by HPV 16 or 18, sensitivity would increase to 66% (2/3), since an HPV 18+ adenosquamous carcinoma was negative by OncoE6. It is difficult to account for the OncoE6 failure to detect one carcinoma containing HPV 18. Histological assessment showed this to be an undifferentiated adenosquamous tumor, which may have been less likely to be driven by HPV 18 E6 synthesis.

In the study of Zhao and co-workers [20], HPV 45 E6 was detectable in addition to HPV 16 and HPV 18 E6. Of note, the rate of OncoE6 and HPV-DNA positive women was similar in both studies i.e. 1.4% and 18.7% in the Amazonas (current study) and 1.8% and 14–18% in China, respectively. Inclusion of HPV 45 E6 in the current version of OncoE6 is feasible given that it is already available in the version used in the Chinese study [21]; and, it would have little impact on test specificity, since only one patient was infected by this genotype among all 412 investigated.

Performance of HPV NAT on the same self-collected sample and referral of all HPV-DNA+ to colposcopy, afforded insight into the incidence of CC and precursor lesions in the population investigated. PCR screening would certainly provide optimal sensitivity, but -in addition- to the aforementioned challenges, would also be technically complex under local conditions. Nonetheless, it does provide a reliable gold-standard to estimate OncoE6 clinical sensitivity.

A recent study [21] also employing OncoE6 for CC screening corroborated a specificity of 99.1%. However, on re-screening all initially positive women one year later, it was observed that OncoE6 poorly predicted CIN2+ incidence, rendering it an excellent diagnostic tool–albeit- inefficient for CC screening. This suggests that if OncoE6 is to be adopted as a screening tool in the Amazon population, it would require more frequent testing than the large intervals of> 5 years currently afforded by HPV DNA screening. As suggested by Valdez et al.[21], decreasing the limit of detection of E6 protein and including HPV 45 E6 and possibly other high-risk types may increase clinical sensitivity and improve the negative predictive value of OncoE6, which could then be used as an isolated screening strategy in populations with limited access to HPV-NAT. Eventually, we foresee a strategy of providing self-collection+OncoE6 to all under/unscreened *ribeirinhas*, with a view toward detection of the majority of the CIN3+ cases. This could further move to self-collection+DNA screening/OncoE6 strategy by capacitating regional labs to implement automated NATs. As a model to be replicated, for several years, HIV viral load has been performed locally in all Brazilian states [22], drawing on a network of public laboratories. Alternatively, simpler hrHPV DNA NAT like the careHPV (Qiagen,) could be used for screening and OncoE6 used for triage, as demonstrated in some studies [15,20]. Both methods may be performed with the same self-collected sample and testing performed locally.

The strategy here presented has not been evaluated for its cost-effectiveness. However, the costs were evaluated and compared to those incurred for transportation and treatment of women with ICC. Of note, most are diagnosed at FIGO grade II or higher (FCECON unpublished data). When combined with the social and economic costs of the loss of women in economic productive age, it will most likely prove to be cost-effective.

Despite boasting the highest burden of ICC in Latin America, there seems to be reluctance to adopt new strategies for CC screening in Brazil with continued advocacy for the use of the Pap test. In contrast in Argentina [23] and several other Latin American countries [24] a similar approach of self-sampling at home visits and further HPV-DNA testing by Hybrid Capture 2 was successfully introduced in the Jujuy province.

Our study mobilized many key stakeholders in CC prevention and treatment in the Amazonas state. Many are convinced by the results and the feasibility of this strategy. There are plans to expand the current approach to approximately 10,000 *ribeirinhas*, while establishing permanent centers, locally, for colposcopy and treatment of precursor lesions. We believe that, by continuously promoting and improving this program, CC incidence in the Amazonas state may decline within a few years. In conjunction to HPV vaccination, which is routine for girls since 2013 in the state and was extended to boys, in the whole country, from 2017 on, eradication of CC seems an achievable goal in the next two decades.
